# Critical Aspects into Surgical Management, Classification, and Therapeutic Guidelines for Incidental GISTs During Bariatric Surgeries

**DOI:** 10.1007/s11695-025-07919-0

**Published:** 2025-05-19

**Authors:** Hassan El-Masry, Mohamed Abouegylah, Ahmed Abokhozima

**Affiliations:** 1 Alexandria University, Alexandria, Alexandria, Egypt, Egypt; 2 El-Ekbal Hospital, Alexandria, Alexandria, Egypt, Egypt

**Keywords:** GISTs, Resectional bariatric surgery, Imatinib, NTRK fusion-positive

## Abstract

Incidental gastrointestinal stromal tumors (GISTs) discovered during bariatric surgeries present unique diagnostic and management challenges. In response to Khan et al.’s report on a sleeve-preserving approach to GIST resection, we highlight several critical considerations. Contrary to the notion that incidental GISTs rarely alter surgical plans, tumor characteristics, especially size and location near critical structures like the gastroesophageal junction, can necessitate procedural modifications. Preoperative endoscopy plays a pivotal role in early detection and surgical planning. We also underscore the importance of individualized oncologic decision-making, integrating tumor parameters and procedural classification systems.

Dear Editor,

We commend Khan et al. for their insightful article, “Localized Excision of Gastrointestinal Stromal Tumor (GIST) After Sleeve Gastrectomy: Highlighting a Sleeve-Preserving Surgical Approach.” The case report provides valuable insights into the management of GISTs in post-sleeve gastrectomy patients and underscores the importance of preoperative evaluation and long-term follow-up [1]. The authors’ emphasis on a sleeve-preserving approach is particularly noteworthy, as it highlights the balance between oncologic safety and maintaining gastric integrity. However, we would like to raise a few critical points for further discussion.

Firstly, the authors state that “When GISTs are incidentally found during bariatric surgery, resection is often feasible without altering the original surgical plan, and complete resection with negative margins is usually curative [1].” While this is generally true for small, low-risk tumors, it is important to acknowledge that discovering a GIST during bariatric surgery can alter the surgical plan [2]. For instance, tumors located near critical structures such as the gastroesophageal junction (GEJ) or those requiring extensive resection may necessitate modifications to the original procedure.

Additionally, the role of preoperative upper gastrointestinal endoscopy in detecting such lesions cannot be overstated [3]. As demonstrated in by Khan et al., endoscopic evaluation revealed a polypoidal mass at the posteromedial aspect of the GEJ along the lesser curvature, with endoscopic ultrasound (EUS) confirming its origin from the muscularis propria [1].

In such cases, the surgeon must weigh the risks of preserving the sleeve against the need for complete oncologic resection. This case report demonstrates this complexity, as the tumor’s location at the GEJ required careful mobilization and a tailored approach to preserve the sleeve [1]. This nuance underscores the importance of avoiding oversimplification in the decision-making process for such scenarios, a point emphasized in a review by Abokhozima et al. [2].

The decision-making process is further complicated by the variability in tumor size, location, and malignant potential. For tumor size, Fernández et al. recommend that lesions >2 cm should be resected with or without bariatric surgery, while smaller tumors (<2 cm) may be managed with endoscopic follow-up or resection during bariatric surgery [4]. In the case reported by Khan et al. [1], where a GIST was identified after sleeve gastrectomy via upper endoscopy (not incidentally during the procedure), a sleeve-preserving approach with complete oncologic resection was feasible but required meticulous planning to avoid compromising gastric function or oncologic outcomes.

Zidan et al. recently proposed a refined parametric classification of metabolic and bariatric surgeries (MBS), categorizing procedures into resectional (RBS) and non-resectional (NRBS) approaches to better reflect anatomical and functional outcomes. This framework further subdivides RBS into isolate gastric, gastrointestinal bypass, and isolate intestinal bypass surgeries, emphasizing the role of gastric resection in enabling thorough intraoperative exploration and histopathological evaluation [5]. Their work highlights that RBS, such as sleeve gastrectomy (SG) or resectional gastric bypass (RGB), offers distinct advantages in managing incidental findings like GISTs, as resection allows direct access to the gastric wall and facilitates complete tumor excision without compromising oncologic margins. For instance, Zidan et al. reported a 100% modification rate in surgical plans for Roux-en-Y gastric bypass (RYGB) cases where incidental GISTs were identified in the gastric remnant, underscoring the critical need for adaptable strategies within the RBS paradigm [2, 5].

The likelihood of detecting incidental GISTs varies by surgical approach. RBS, such as sleeve gastrectomy, enable thorough gastric inspection, improving incidental detection rates. In contrast, non-resectional procedures (NRBS) limit gastric exposure, potentially delaying diagnosis. This disparity underscores the value of RBS in high-risk populations and the need for systematic histopathologic evaluation of resected specimens [5].

In terms of post-operative histopathology, Khan et al. also align with the broader literature emphasizing its importance Incidental GISTs are often diagnosed post-operatively [1], as seen in 22.5% of cases in the review by Abokhozima et al. [2], underscoring the value of routine histopathological evaluation after resectional bariatric surgeries. This practice not only ensures complete tumor removal but also contributes to long-term oncologic safety and patient outcomes [6].

Secondly, the authors state that “Larotrectinib and Entrectinib are the preferred first-line therapies for individuals with GISTs that are unresectable, advanced, or have metastasized” [1]. Per the National Comprehensive Cancer Network (NCCN) 2024 guidelines, imatinib, targeting KIT and PDGFRA mutations, remains the first-line standard for most advanced GISTs, except for PDGFRA D842V-mutant tumors (treated with avapritinib). Larotrectinib and entrectinib are reserved exclusively for the rare (<1%) NTRK fusion-positive subset, which the authors do not explicitly mention [1, 7]. By omitting these disparities, the study risks misguiding clinicians unfamiliar with GIST’s molecular heterogeneity [7].

While Khan et al. provide a valuable contribution to the literature on GIST management in post-bariatric surgery patients, these points warrant further clarification to ensure accurate and comprehensive guidance for bariatric surgeons.

## Data Availability

No datasets were generated or analysed during the current study.
